# Expressions of glycemic parameters, lipid profile, and thyroid hormone in patients with type 2 diabetes mellitus and their correlation

**DOI:** 10.1002/iid3.1282

**Published:** 2024-07-05

**Authors:** Hua Chen, Jing Wu, Rui Lyu

**Affiliations:** ^1^ Department of Endocrinology Pu'er People's Hospital Pu'er Yunnan Province PR China

**Keywords:** correlation, glycolipid metabolism, T2DM, thyroid hormone

## Abstract

**Objective:**

This study aimed to investigate the expressions of glycemic parameters, lipid profile, and thyroid hormone in type 2 diabetes mellitus (T2DM) patients and their correlation.

**Methods:**

Eighty‐four patients with T2DM in our hospital were included as the observation group. The T2DM patients were divided into mild group, moderate group, and severe group according to the fasting plasma glucose (FPG) level. Another 84 healthy subjects in the same period of health examination in our hospital were included as the control group. The levels of glycemic parameters, (HbA1c and FPG), lipid profile (TC, TG, LDL‐C, and HDL‐C) and thyroid hormone (FT3, TSH, and FT4) were measured by automatic biochemical analyzer. The correlation between glycemic parameters, lipid profile, and thyroid hormone was analyzed by Pearson correlation analysis.

**Results:**

The FPG, TC, TG, LDL‐C, HbA1c, and TSH levels were significantly elevated, while the HDL‐C and FT3 levels were significantly declined in the observation group versus to control group (*p* < .05). The levels of HbA1c, FPG, TC, LDL‐C, and TSH were significantly increased, while the levels of HDL‐C and FT3 were decreased in moderate and severe groups, when compared to mild group (*p* < .05). The levels of HbA1c, FPG, TC, LDL‐C and TSH were higher, while the level of FT3 was lower in severe group than those in moderate group (*p* < .05). Pearson Correlation analysis showed that FT3 level in T2DM patients was positively correlated with FPG, HbAlc, TC, TG, and LDL‐C levels (*p* < .05), but negatively correlated with HDL‐C level (*p* < .05). TSH level was negatively correlated with FPG, HbAlc, TC, TG, and LDL‐C levels (*p* < .05), while positively correlated with HDL‐C level.

**Conclusion:**

The thyroid hormone levels were of clinical significance in evaluating glycolipid metabolism and severity of T2DM. Clinical detection of glycolipid metabolism and thyroid hormone levels in T2DM patients is of great significance for diagnosis, evaluation, and targeted treatment of the disease.

## 1 INTRODUCTION

With the improvement of people's living standards, the intake of plasma glucose and lipid of Chinese residents has increased year by year, resulting in an increasing number of diabetes patients, especially in the elderly group, with a significant increase in incidence.^1^ Diabetes is a common disease with a very high incidence in China, and has become one of the chronic diseases that seriously affect the health of patients.^2^, ^3^ Clinically, diabetes is divided into type 1 diabetes and type 2 diabetes mellitus (T2DM), in which T2DM is a chronic progressive metabolic disease, which is a series of immune responses caused by the lack of insulin, leading to increased inflammation, which leads to the disorder of glucose and lipid metabolism.^4^, ^5^ Hyperglycaemia and hyperlipidemia in T2DM interact with each other, that is, hyperglycaemia causes lipid metabolism disorders, and the glycerol produced from lipolysis is converted into glucose through a glycolytic metabolic pathway. Therefore, controlling the plasma glucose and lipid intake should be taken as an important measure to prevent and treat the complications of diabetes.

The thyroid diseases are autoimmune diseases, and the changes in the internal environment of diabetic patients affect the levels of thyroid hormone, which in turn affects the metabolism of glucose and lipid.^6^, ^7^ Previous studies have shown that the incidence of thyroid dysfunction and dyslipidemia in diabetic population is significantly increased, which is 2‐3 times that of nondiabetic population.^8^, ^9^ Ozair et al. found that the prevalence of thyroid dysfunction in patients with T2DM was 28%, and subclinical hypothyroidism (SCH) was the main one, up to 18.8%.^10^ Han et al. collected a large number of studies on T2DM combined with SCH and conducted a meta‐analysis, which showed that the probability of SCH in T2DM patients was 10.2%, and SCH also affected the occurrence of diabetes complications.^11^ Moreover, increasing evidence have demonstrated that patients with T2DM may affect their thyroid function to varying degrees through multiple pathways, and thyroid dysfunction also has adverse effects on metabolism, which accelerates the clinical process of T2DM.^12^, ^13^ Therefore, T2DM patients should be regularly screened for thyroid function, and conversely, patients with thyroid dysfunction should also be regularly monitored plasma glucose and lipids, which can prevent and diagnose diabetes in an early stage.

This study aimed to investigate the expressions of glycolipid metabolism and thyroid hormone in T2DM patients and their correlation, which will provide data support for clinical prevention and treatment of T2DM.

## 2 METHODS

### 2.1 Participants

A total of 84 patients diagnosed with T2DM who were admitted to the hospital's endocrinology department from July 2019 to May 2021 were included as the observation group, meanwhile, 84 patients in the same period of health examination in the hospital were included as the control group.

Inclusion criteria: The relevant diagnostic criteria in the Chinese Guidelines for the Prevention and Treatment of Type 2 Diabetes (2013 edition)^14^ were met; The data were complete; There were no acute complications of diabetes. Exclusion criteria: Patients with acute complications of diabetes; Patients with severe cardiopulmonary and liver and kidney diseases had received drug treatment in the past 6 months; Patients with hypoparathyroidism or hyperactivity; Patients with malignant tumors; Patients with other endocrine and metabolic diseases; Patients with critical illness needing ICU admission. This study was approved by the Ethics Committee of our hospital, and all subjects signed informed consent.

### 2.2 Collection of baseline data

General clinical data, such as gender, age, smoking history, alcohol consumption, height, and weight, were recorded through review of patients' medical records.

### 2.3 Examination of body indices

The patient's height and weight were measured by trained medical personnel using standard measurement methods. On the day of admission, the height and weight were measured in the fasting state with minimum clothing and without shoes. Body mass index (BMI) = weight/height squared.

### 2.4 Measurement of glucose and lipid metabolism levels

All subjects were forbidden to eat at night, and fasting median elbow venous plasma was collected in the morning of the next day. The collected plasma was immediately transported to the central laboratory of our hospital. Serum was isolated and stored at −80°C for use. The level of HbA1c was performed by high performance ion exchange chromatography method using ZellBio GmbH Kit (Ulm). Fasting plasma glucose (FPG), TC, TG, and HDL‐c levels were measured by routine enzymatic assays with commercial kit (Pars Azmoon) using an autoanalyzer (AVIDA 1800 chemistry system; Siemens). All laboratory measurements were performed in the laboratory of Diabetes Research Center utilizing the standard methods.

### 2.5 Measurement of thyroid hormone levels

Serum levels of TSH, FT3, and FT4 were measured by electrochemiluminescence (Roche) according to the manufacturer's introduction.

### 2.6 Statistical analysis

The data were analyzed using SPSS 22.0 software (IBM). All data were tested for normality by Shapiro–Wilk normality test. Continuous variables that conform to a normal distribution are expressed as mean ± standard deviation. The differences between two groups were compared by Student's *t*‐test. Categorical variables were expressed as percentages and comparison of two groups was performed using *χ* ^2^ test. One‐way analysis of variance followed by post hoc tests was used for comparison among the multiple groups. The relationship between glycolipid metabolism and thyroid hormone levels were analyzed by Pearson correlation analysis. A value of *p* < .05 indicated the difference was statistically significant.

## 3 RESULTS

### 3.1 Comparison of clinical data between the two groups

A total of 84 T2DM patients were included as observation group, and 84 healthy subjects were included as control group. The basic information such as age, gender, smoking, drinking, height, and weight of all subjects were recorded. Results discovered that the differences between the observation and control groups in terms of age, gender, smoking, and drinking were not significant (*p* > .05). But, the BMI in observation group was significant higher than that in control group (*p* < .05) (Table 1).

**Table 1:** Comparison of clinical data between the two groups.

Project	Control group (*n* = 84)	Observation group (*n* = 84)	*t*/*χ* ^2^	*p*
Age (year)	59.35 ± 6.92	58.34 ± 6.57	0.970	.333
Gender (male/female, n/n)	46/38	50/34	0.389	.533
Smoking (yes/no, n/n)	44/40	48/36	0.384	.535
Drinking (yes/no, n/n)	39/45	43/41	0.381	.537
BMI (kg/m^2^)	20.45 ± 3.12	21.47 ± 3.24*	2.08	.039

Abbreviation: BMI, body mass index.

* *p* < .05 versus control group.

### 3.2 Measurement of glycolipid metabolism and thyroid hormone levels in two groups

Then, the glycolipid metabolism and thyroid hormone levels were measured in the two groups. As shown in Table 2, the levels of HbA1c, FPG, TC, TG, and LDL‐C were obviously increased, while the levels of HDL‐C were decreased in observation group, when compared to control group (*p* < .05). Besides which, compared with control group, the levels of FT3 were signally decreased, while TSH levels were significantly increased in observation group (*p* < .05). Moreover, there was no significant difference in FT4 levels between the two groups (*p* > .05) (Table 3).

**Table 2:** Measurement of glycolipid metabolism levels in the two groups.

Group	Cases	HbA1c (%)	FPG (mmol/L)	TC (mmol/L)	TG (mmol/L)	LDL‐C (mmol/L)	HDL‐C (mmol/L)
Control group	84	4.48 ± 0.69	5.04 ± 0.61	4.46 ± 0.68	1.42 ± 0.25	2.40 ± 0.35	1.68 ± 0.18
Observation group	84	7.16 ± 1.52*	8.98 ± 2.55*	5.47 ± 0.84*	2.19 ± 0.47*	3.15 ± 0.49*	1.20 ± 0.28*
*t*		14.71	13.773	8.565	13.257	11.415	13.216
*p*		<.001	<.001	<.001	<.001	<.001	<.001

Abbreviations: FPG, fasting plasma glucose; HbA1c, hemoglobin A1c; HDL‐c, high density lipoprotein‐cholesterol; LDL‐c, low density lipoprotein‐cholesterol; TC, total cholesterol; TG, triglyceride.

* *p* < .05 versus control group.

**Table 3:** Measurement of thyroid hormone levels in the two groups.

Group	Cases	FT3 (pmol/L)	TSH (μIU/mL)	FT4 (pmol/L)
Control group	84	4.15 ± 0.99	2.25 ± 0.78	16.02 ± 2.12
Observation group	84	3.59 ± 0.85*	2.77 ± 0.87*	15.91 ± 1.72
*t*		3.933	4.079	0.369
*p*		<.001	<.001	.712

Abbreviations: FT3: free triiodothyronine; TSH: thyroid stimulating hormone; FT4: free thyroxine.

* *p* < .05 versus control group.

### 3.3 Measurement of glycolipid metabolism and thyroid hormone levels in different degrees of T2DM patients

Subsequently, we measured the levels of glycolipid metabolism and thyroid hormone in different degrees of T2DM patients. Results showed that the levels of HbA1c, FPG, TC, LDL‐C, and TSH in moderate and severe groups were significantly increased, while the levels of HDL‐C and FT3 were decreased when compared to mild group (*p* < .05). The levels of HbA1c, FPG, TC, LDL‐C, and TSH in severe group were higher, while the levels of FT3 were lower than those in moderate group (*p* < .05). However, there was no significant difference in FT4 among the three groups. Moreover, the levels of HDL‐C in severe group and moderate group have no significant differences (*p* > .05) (Table 4 and 5).

**Table 4:** Measurement of glycolipid metabolism levels in different degrees of T2DM patients.

Group	Cases	HbA1c (%)	FPG (mmol/L)	TC (mmol/L)	TG (mmol/L)	LDL‐C (mmol/L)	HDL‐C (mmol/L)
Mild group	41	5.92 ± 0.72	6.76 ± 0.72	4.82 ± 0.34	1.84 ± 0.26	2.84 ± 0.32	1.42 ± 0.22
Moderate group	30	7.92 ± 0.84*	10.29 ± 1.13*	5.84 ± 0.63*	2.42 ± 0.31*	3.36 ± 0.41*	1.03 ± 0.14*
Severe group	13	9.34 ± 0.91* ^,^ **	12.97 ± 1.34* ^,^ **	6.64 ± 0.55* ^,^ **	2.76 ± 0.42* ^,^ **	3.62 ± 0.48* ^,^ **	0.95 ± 0.12*
*F*		112.584	235.151	80.376	57.671	28.070	55.346
*p*		<.001	<.001	<.001	<.001	<.001	<.001

Abbreviations: FPG, fasting plasma glucose; HbA1c, hemoglobin A1c; HDL‐c, high density lipoprotein‐cholesterol; LDL‐c, low density lipoprotein‐cholesterol; TC, total cholesterol; TG, triglyceride.

* *p* < .05 versus mild group;

** *p* < .05 versus moderate group.

**Table 5:** Measurement of thyroid hormone levels in different degrees of T2DM patients.

Group	Cases	FT3 (pmol/L)	TSH (μIU/mL)	FT4 (pmol/L)
Mild group	41	3.97 ± 0.84	2.34 ± 0.64	16.04 ± 1.85
Moderate group	30	3.46 ± 0.65*	2.94 ± 0.77*	15.86 ± 1.64
Severe group	13	2.72 ± 0.56* ^,^ **	3.74 ± 0.84* ^,^ **	15.68 ± 1.59
*F*		14.891	19.920	0.239
*p*		<.001	<.001	.788

Abbreviations: FT3, free triiodothyronine; FT4, free thyroxine; TSH, thyroid stimulating hormone.

* *p* < .05 versus mild group;

** *p* < .05 versus moderate group.

### 3.4 Correlation analysis of glycolipid metabolism and thyroid hormone

Table 6 showed the Pearson correlation coefficients between glycolipid metabolism and thyroid hormone in patients with T2DM. Results revealed that FT3 levels in T2DM patients were positively correlated with FPG, HbAlc, TC, TG, and LDL‐C levels (*p* < .05), but negatively correlated with HDL‐C (*p* < .05). TSH levels were negatively correlated with FPG, HbAlc, TC, TG, and LDL‐C levels (*p* < .05), while positively correlated with HDL‐C. There was no correlation between FT4 and the above indexes (*p* > .05).

**Table 6:** Correlation between thyroid hormone and glycolipid metabolism levels in T2DM patients.

Index	FT3		TSH		FT4	
	*r*	*p*	*r*	*p*	*r*	*p*
HbA1c (%)	−0.38	<.01	0.52	<.01	−0.12	.29
FPG (mmol/L)	−0.49	<.01	0.53	<.01	−0.05	.66
TC (mmol/L)	−0.47	<.01	0.44	<.01	0.08	.47
TG (mmol/L)	−0.39	<.01	0.43	<.01	−0.06	.58
LDL‐C (mmol/L)	−0.32	<.01	0.28	.01	−0.05	.65
HDL‐C (mmol/L)	0.42	<.01	−0.44	<.01	0.02	.86

Abbreviations: FPG, fasting plasma glucose; FT3, free triiodothyronine; FT4, free thyroxine; HbA1c, hemoglobin A1c; HDL‐c, high density lipoprotein‐cholesterol; LDL‐c, low density lipoprotein‐cholesterol; TC, total cholesterol; TG, triglyceride; TSH, thyroid stimulating hormone.

## 4 DISCUSSION

T2DM is a chronic inflammatory disease characterized by systemic metabolic disorders, with multiple pathogenic factors, which leads to the increase of plasma glucose and the disorder of protein and other substances in the body, resulting in cardiovascular diseases and other complications, seriously threatening human health.^15^ Therefore, early diagnosis and reasonable treatment are important countermeasures to delay the development of T2DM, which is of great significance to save patients' life andimprove their quality of life.

Many T2DM patients are complicated with multiple cardiovascular diseases. With the increase of risk factor, the prevalence of lipid metabolism disorders, hypertension, and other diseases also presents an increasing trend.^16^ Jiang et al. found that the disorder of glucose and lipid metabolism indexes was a risk factor for the onset of T2DM, which accelerated the release of inflammatory factors, eventually leading to the worsening of the disease.^17^ TG, TC, HDL‐C, and LDL‐C reflect the lipid metabolism of the body, and their levels are disturbed when T2DM occurs.^18^ FPG and HbA1c are commonly used to detect diabetes, which reflect the plasma glucose levels of the body.^19^ A previous study revealed that T2DM patients were more prone to changes in lipid profile of arteriosclerosis, which was specifically manifested as significantly increased levels of TC, TG, and LDL‐C.^20^ Another study demonstrated that hypertriglyceridemia acted as an independent predictor of diabetes incidence.^21^ In this study, we found that the levels of HbA1c, FPG, TC, TG, and LDL‐C were higher, while the levels of HDL‐C were lower in the observation group than those in control group, which was in line with the above studies. In addition, the levels of HbA1c, FPG, TC, TG, and LDL‐C were lower, while the levels of HDL‐C were higher in the mild group than those in the moderate and severe groups, suggesting that the T2DM patients were mostly accompanied by abnormal glucose and lipid metabolism, and the more severe the disease, the more serious the disorder of glucose and lipid metabolism. Therefore, the plasma glucose and lipid levels of T2DM patients should be tested regularly to accurately assess the condition and then develop a reasonable treatment plan to improve the treatment effect.

Increasing studies have suggested that in addition to the disorder of glucose metabolism, T2DM patients are often accompanied by immune thyroid dysfunction.^22^, ^23^ Demitrost et al. found that the incidence of thyroid dysfunction in T2DM patients was 31.2%, of which 16.3% was SCH.^24^ Roa Dueñas et al. revealed that people with hypothyroidism and FT4 levels at low normal levels had an increased risk of developing T2DM.^25^ Moreover, previous studies demonstrated that FT3 levels were lower in T2DM patients than that in healthy people, indicating that FT3 might be associated with the incidence of T2DM.^26^, ^27^ Another study discovered that high‐normal TSH levels were correlated with increased incidence of advanced fibrosis in T2DM patients.^28^ Besides, a study displayed that high‐normal TSH levels caused an increase in FPG, TC, TG, and LDL‐C, which were associated with T2DM development.^29^ In addition, it has been shown that hypothyroidism was associated with hypertriglyceridemia,^30^ which was a strong risk factor for T2DM.^31^ These results were consistent with our study that lower FT3 and higher TSH levels were found in the observation group, when compared with the control group. In addition, our study also showed that FT3 levels in the mild group were higher than those in the moderate and severe groups, and TSH levels were lower than those in the moderate and severe groups, indicating that SCH may be the main factor that causes T2DM. In addition, we found that FT3 levels were positively, while TSH levels were negatively correlated with FPG, HbAlc, TC, TG, and LDL‐C in T2DM patients, suggesting that the disorder of glucose and lipid metabolism in T2DM patients may cause changes in thyroid hormone secretion, and the reduction of TSH transport ability will inhibit its secretion function, and then lead to the decrease of FT3 levels.^32^, ^33^, ^34^ Therefore, thyroid function and lipid levels in T2DM patients should be diagnosed early, and timely intervention should be performed if abnormalities are found.

There are some limitations in this study. Firstly, the sample size is relatively small, which may lead to bias in the results, and it is necessary to further increase the sample size in later studies. Secondly, this study is a retrospective study, which has certain limitations, such as poor data quality, and can't determine the causal relationship between hypothyroidism and T2DM. Thirdly, the effects of hypoglycemic drugs, lipid‐regulating drugs and other chronic complications on thyroid hormone levels were not considered. Moreover, the differences in analysis methods were not considered, and require further work is required in the further.

## 5 CONCLUSION

The levels of glycemic parameters, lipid profile, and thyroid hormone in T2DM patients were significantly different from those of healthy subjects, and their levels change continuously with the exacerbation of patients' disease. People with SCH and abnormal glucose and lipid metabolism may be at significantly increased risk of T2DM, which requires clinical attention and effective prevention and treatment measures.

## AUTHOR CONTRIBUTIONS

Rui Lyu and Jing Wu designed and supervised the entire study, and helped with critical reading of the manuscript. Hua Chen performed the experiments, made significant contributions to data acquisition and analysis, and performed the manuscript draft. All authors contributed to editorial changes in the manuscript. All authors read and approved the final manuscript. All authors have participated sufficiently in the work and agreed to be accountable for all aspects of the work.

## CONFLICT OF INTEREST STATEMENT

The authors declare no conflict of interest.

## Data Availability

The datasets used and/or analyzed during the present study are available from the corresponding author on reasonable request.
